# QUADRICEPS STRENGTH IS ASSOCIATED WITH COGNITION IN OLDER ADULTS WITH CHRONIC STROKE

**DOI:** 10.1093/geroni/igz038.2431

**Published:** 2019-11-08

**Authors:** Kimberly Bennett, Rachel A Crockett, Lisanne F ten Brinke, Jennifer C Davis, Teresa Liu-Ambrose

**Affiliations:** 1 University of British Columbia, Vancouver, Canada; 2 University of British Columbia, Vancouver, British Columbia, Canada; 3 University of British Columbia - Okanagan Campus, Kelowna, British Columbia, Canada

## Abstract

Individuals who have suffered a stroke are at risk for developing cognitive impairment and dementia. Thus, it is important to identify modifiable risk factor for cognitive decline in this population. Among older adults without a history of stroke, greater muscle strength is associated with better cognitive function. Whether this relationship also exist in older adults with a history of stroke is not known. Thus, we aimed to examine whether cognition, as measured by both the Montreal Cognitive Assessment (MoCA) and the 13-item Alzheimer’s Disease Assessment Scale-Cognitive (ADAS-Cog 13), is associated with lower extremity muscle strength in adults with chronic stroke (> 1 year post stroke). Ninety-one community-dwelling adults, aged 55 years and older, with chronic stroke were included in this analysis. Isometric strength of the quadriceps was measured bilaterally in kilograms. Two linear regression models were constructed to determine the independent association of quadriceps strength (mean kilograms of both legs) with: 1) MoCA; and 2) ADAS-Cog 13, after controlling for age, sex, and mood. Mean quadriceps strength was independently associated with both MoCA and ADAS-Cog scores, after accounting for age, sex, and mood. Specifically, quadriceps strength explained an additional 5.6% of the variable in MoCA scores; total variance explained by the model was 12.0%. For ADAS-Cog 13, quadriceps strength explained an additional 5.4% of the variance; total variance explained by the model was 16.5%. Our current cross-sectional results suggest that the maintenance of muscle strength may be important for cognitive health in older adults who have suffered a stroke.

